# High throughput in vitro characterization of pectins for pig(let) nutrition

**DOI:** 10.1186/s42523-021-00129-w

**Published:** 2021-10-09

**Authors:** Maria Wiese, Yan Hui, Jesper Holck, Jimmy J. P. Sejberg, Celia Daures, Evy Maas, Witold Kot, Johanna M. Borné, Bekzod Khakimov, Thomas Thymann, Dennis Sandris Nielsen

**Affiliations:** 1 CP Kelco ApS, Lille Skensved, Denmark; 2 Department of Food Science, University of Copenhagen, Rolighedsvej 26, 1958 Frederiksberg C, Denmark; 3 Section for Protein Chemistry and Enzyme Technology, Department of Biotechnology and Biomedicine, Technical University of Denmark, Kgs. Lyngby, Denmark; 4 Department of Plant and Environmental Sciences, University of Copenhagen, Frederiksberg, Denmark; 5 Department of Veterinary and Animal Sciences, University of Copenhagen, Frederiksberg, Denmark; 6 Present Address: Microbiology and Systems Biology Department, TNO, Utrechtsweg 48, 3704 HE Zeist, The Netherlands

**Keywords:** Pectin, Pectin structure, Piglet, Gut microbiome (GM), Weaning, Short-chain fatty acids (SCFA), In vitro colon simulation, Prebiotic

## Abstract

**Background:**

Fiber-rich feed components possess prebiotic potential to enhance pig health and are considered a potential solution to the high prevalence of post-weaning diarrhea in pig production under the phased suspension of antibiotics and zinc oxide use.

**Methods:**

We screened the gut microbiota modulatory properties of pectin substrates prepared from sugar beet within the freshly weaned piglet gut microbiome using an in vitro colon model, the CoMiniGut. We focused on testing a variety (13) of sugar beet-derived pectin substrates with defined structures, as well as known prebiotics such as inulin, fructooligosaccharide (FOS) and galactooligosaccharide (GOS), to gain insights on the structure–function related properties of specific substrates on the weaner gut microbial composition as well as shortchain fatty acid production (SCFA).

**Results:**

Sugar beet-derived pectin and rhamnogalacturonan-I selectively increased the relative abundance of *Bacteroidetes*, specifically *Prevotella copri, Bacteroides ovatus*, *Bacteroides acidificiens*, and an unclassified *Bacteroides* member. The degree of esterification impacted the relative abundance of these species and the SCFA production during the in vitro fermentations. Modified arabinans derived from sugar beet promoted the growth of *Blautia, P. copri, Lachnospiraceae* members and *Limosilactobacillus mucosae* and amongst all oligosaccharides tested yielded the highest amount of total SCFA produced after 24 h of fermentation. Sugar beet-derived substrates yielded higher total SCFA concentrations (especially acetic and propionic acid) relative to the known prebiotics inulin, FOS and GOS.

**Conclusion:**

Our results indicate that the molecular structures of pectin, that can be prepared form just one plant source (sugar beet) can selectively stimulate different GM members, highlighting the potential of utilizing pectin substrates as targeted GM modulatory ingredients.

**Supplementary Information:**

The online version contains supplementary material available at 10.1186/s42523-021-00129-w.

## Introduction

Stunted growth, high mortality, and the use of antibiotics and zinc oxide due to gastrointestinal dystrophy and diarrhea during the first months of life are ethical and economic problems for piglet producers worldwide [1, 2]. Continuous efforts are thus needed to elucidate how feed components and alternatives to in-feed antibiotics can improve pig health [3]. Options include feed supplementation with probiotic cultures, organic acids, and prebiotic indigestible oligosaccharides to promote intestinal functionality and strengthening of the gut microbial community homeostasis and related associated metabolites such as short-chain fatty acids (SCFA) [4–8].

A biotic gut microbiome (GM) with intrinsic resilience persists perturbations and offers protection from pathogenic bacteria through competitive exclusion [9, 10]. The GM also promotes the development and maturation of the gastrointestinal tract (GIT) and the mucosal system [8, 11]. Diet is a principal factor shaping GM composition and functionality [12]. The addition of fermentable fibre into feed can be especially beneficial, as it can stimulate the development of the indigenous GM and fosters the production of SCFAs. Short-chain fatty acids are the end-products of fibre fermentation by the GM and play important roles in gut health [13, 14]. Short-chain fatty acids serve as a source of energy for the host and act as antimicrobials due to their pH-lowering properties. They can also increase pancreatic secretion and exhibit trophic effects on the gastrointestinal tract [15, 16].

The piglet GM goes through a phase of maturation with the most critical stages of gut colonisation being right after birth and during weaning [17]. In particular, the diversity and capacity of the GM to ferment carbohydrates is still limited at early age [18].

The weaner GM is mainly able to utilize simple carbohydrates and milk oligosaccharides[19], as it has evolved and adapted to feed on porcine milk oligosaccharides (PMOs) present in the sow milk [20]. Oligosaccharides such as FOS (fructooligosaccharides) and GOS (galactooligosaccharides) are currently implemented as replacement oligosaccharides in weaner feed [21], whereas the potential of pectic oligosaccharides (POS) for such applications is still largely unexplored.

Pectin is a major component of all non-lignocellulosic plant cell walls, and it has been hypothesized that the mammalian GIT has co-evolved with and adapted to pectin, as crude plant food has been consumed in the animal kingdom for millions of years. The three major pectic polysaccharides are homogalacturonan (HG), rhamnogalacturonan-I (RG-I) and rhamnogalacturonan-II (RG-II) in varying proportions depending on plant source [22]. The detailed structure of pectin can differ significantly regarding the degree of esterification (DE), molecular weight, and fine branching of sugar structures. Depending on the plant source and extraction method, this structure can be modified by various physicochemical and enzymatic processes [23].

The structure-to-function related mode of action of pectin in the GIT remains poorly understood, partly because the recent advances in sequencing technologies, that facilitate whole microbiome analysis are just starting to be applied to the area of in-feed prebiotics. One example is a study by Tian et al. 2017 who investigated the impact of high methylated citrus pectin (HMP) and low-methylated citrus pectin (LMP) on weaner colon GM showing that HMP supplementation shifted the weaner GM from a *Lactobacillus* dominated GM to a *Prevotella* dominated GM [24].

Sugar beet pulp is a by-product of the sugar-refining industry and has already been implemented in animal feed products in some regions [25–27]. The sugar beet pectin (SBP) displays poorer gelling properties than apple and citrus pectin, widely used in the food industry. SBP differs somewhat in its molecular characteristics due to its comparatively higher content of neutral sugars, a higher degree of acetylation (20–36%), its ferulic acid content, and a higher protein content, in addition to its relatively low molecular mass [28–31]. However, the molecular structure and properties of SBP can vary depending on the extraction methodology [32]. Due to an excess of underutilized industrial sugar beet by-products [32] and the natural history of sugar beet as feed, it makes good sense to explore different extractions and tailor SBP from this source for animal nutrition and health.

Generally, pectin may ameliorate animal health via direct or GM-mediated effects. Various reports exist on the antibacterial and antiviral effects of pectin and its potency to modulate the immune response and the GM. However, the lack of comprehensive insight into the potential of pectin to improve animal growth and health still hampers the development of new, optimised feed mixtures. The potential of pectin to impact pig nutrition and health was recently reviewed [33]. Sugar beet pectins have several potential advantages as weaner fed such as improving growth performance and immunity in weaned pigs challenged by pathogens[33]. In this study, we focus on dissecting the structure to function of sugar beet-derived pectin molecules as substrates for weaned piglet GM in vitro. The aspect of the GM maturity of the animal target group needs to be considered; we hence investigate sugar beet pectin but also related arabinan oligosaccharides for the potential application in weaner feed.

Recent advances in in vitro intestine simulation models [34, 35], and the adaptation of these for the simulation of the piglet intestine, facilitate the development of new platforms for the investigation of feed formulations and their effects on GM composition and related metabolites. In this study we implement the CoMiniGut, a high throughput, low volume in vitro colon model [34], to screen the effects of different pectin substrates and prebiotics on the GM and its SCFA production of freshly weaned piglets in vitro.

## Material and methods

### Preparation of low methylated pectins

Unstandardized sugar beet pectin (SBP) (CP Kelco) was dissolved in deionized water and treated with pectin methylesterase (PME) to achieve the desired degrees of esterification (DE) and esterification patterns (blocky/random). A solution of 0.625 M aqueous ammonia was added continuously using a TIM 856 Titration Manager (Radiometer Analytical) to maintain the pH at the optimum value for the specific PME in use. After complete reaction, the enzymes were inactivated by lowering the pH of the mixtures to 2.5 by addition of aqueous nitric acid followed by heat treatment for 10 min at 80 °C. The pectin solutions were then allowed to cool to approx. 50 °C and pectin was precipitated by pouring the mixtures into 80% aq. 2-propanol. The precipitated pectin was isolated by filtration, washed twice with 60% aq. 2-propanol, dried overnight at 65 °C, milled, and sieved to mesh 60 (DIN 24; 250 μm).

All other substrates used were either CP Kelco products or bought from commercial suppliers, as indicated in Table 1 (where abbreviations of the different substrates used also can be found).

**Table 1:** Substrates used for in vitro fermentations with freshly weaned piglet colon content

Substrates	DE%	DAc%	MW, DP	Sugar composition (mol%)	IV (dL/g)	Ferulic acid (µmol/g DM)	Supplier
1. Sugar Beet Pulp (SBPulp) (dried, 1 mm particle size)	n.d	n.d	I	GalA: 31.2, Rha: 2.7, Ara: 26.9, Gal: 7.0, other:32.2	n.d	41.7 ± 1.6	CP Kelco
2. Sugar Beet Pectin (SBP)*	52	21	69 kDa	GalA: 71.9, Rha: 4.8, Ara: 6.6, Gal: 13.5, other:3.1	2.8	50.5 ± 3.0	CP Kelco
3. RG-I Sugar Beet (SBP-RG-I)*							CP Kelco
4. Sugar Beet LM 1B, Random (LM1B)	44.8	20.1	175 kDa	GalA: 73	2.6	n.d	CP Kelco
5. Sugar Beet LM 1C, Random (LM1C)	37.8	20.3	183 kDa	GalA: 71	2.5	n.d	CP Kelco
6. Sugar Beet LM 1D, Random (LM1D)	22.9	20.4	101 kDa	GalA: 69	2.2	n.d	CP Kelco
7. Sugar Beet LM 2, Blocky (LM2)	35.1	20.5	54 kDa	GalA: 69	1.4	n.d	CP Kelco
8. Sugar Beet LM 2A, Blocky (LM2A)	44.4	20.5	124 kDa	GalA: 73	2.1	n.d	CP Kelco
9. Arabino-oligosaccharides (ModAr)	n.d	n.d	DP 2-15	GalA: 10.4, Rha: 2.5, Ara: 75.1, Gal: 9.6, other:2.4	n.d	52.5 ± 2.9	Holck 2011
10. Arabinan (Ar) from Sugar Beet	n.d	n.d	49 kDa	GalA: 10.2, Rha: 1.4, Ara: 69.0, Gal: 18.7, other:0.7	n.d	n.d	Megazyme
11. Debranched Arabinan (DebAr)	n.d	n.d	28 kDa	GalA: 6.0, Rha: 2.0, Ara: 88, Gal: 4	n.d	n.d	Megazyme
12. Polygalacturonic Acid (C-PG)	n.d	n.d	PI, 13 kDa		n.d	n.d	Sigma Aldrich
13. JEH modified Pectin from Sugar Beet: enriched RGI fraction from sugar beet (ModSBP)	n.d	n.d	50 kDa	GalA: 49.4, Rha: 7.5, Ara: 11.7, Gal: 16.5, other:15.0	n.d	n.d	Holck 2011 tailored
14. Citrus HMP (C-HMP)*							
15. RG-I Citrus (C-RG-I)*	n.d	n.d			n.d	n.d	CP Kelco
16. Inulin from chicory (Inulin)	n.d	n.d	PI, 2 kDa		n.d	n.d	Sigma Aldrich
17. Fructo-oligosaccharides (FOS) (Orafti P95; Oreye, Belgium)	n.d	n.d	DP 2-13		n.d	n.d	Beneo-Orafti
18. Galacto-oligosaccharides (GOS)	n.d	n.d	DP 2–8		n.d	n.d	Beneo-Orafti
19. Pea Pectin (PP)	n.d	n.d			n.d	n.d	CP Kelco
20. Arabinogalactan (AG)	n.d	n.d			n.d	n.d	Sigma-Aldrich

All fermentations tested with 1% (w/v) substrate except were indicated with *, where 0.5% (w/v) was also tested

Molecular structure details such as degree of esterification (DE), degree of acetylation (DAc), molecular weight (MW), degree of polymerization (DP), intrinsic viscosity (IV) and the sugar composition is also listed (abbreviations: *n.d.* not determined, *LM* low methylated, *HMP* high methylated citrus pectin, *RG* rhamnogalacturonan, *GalA* galacturonic acid, *Rha* rhamnose, *Ara* arabinose, *Gal* galactose, *S* soluble, *IN* insoluble, *PI* partly insoluble)

### Analysis of monosaccharide composition

Monosaccharides were obtained by acid hydrolysis according to Sluiter et al. [36] with modifications. All samples were lyophilized prior to hydrolysis. Insoluble substrates were treated in 72% sulfuric acid for 1 h at 30 °C, followed by dilution to 4% sulfuric acid and autoclaved for 1 h at 121 °C. Soluble substrates were dissolved in 4% sulfuric acid and autoclaved for 1 h at 121 °C. All samples were immediately cooled and stored at 4 °C until further use. Monosaccharides were measured by a Dionex High-Performance Anion-Exchange Chromatography with Pulsed Amperometric Detection (HPAEAC-PAD) system equipped with a PA1 column and post-column NaOH addition as described by Zeuner et al. [37].

### Ferulic acid analysis

Ferulic acid was released from substrates by saponification of 150 mg sample in 5 mL 2 M NaOH under N_2_ in the dark overnight. As internal standard, 30 µM cinnamic acid was added before saponification. After saponification, ferulic acid was extracted in 3 × 3 mL ethyl acetate, evaporated under N_2_, and resolubilized in 50% acetonitrile:water before analysis. Quantification of ferulic acid was performed by liquid chromatography-electrospray ionization mass spectrometry (LC–ESI–MS) on an Amazon SL ion-trap (Bruker Daltonics, Bremen Germany) coupled to an UltiMate 3000 UHPLC (Dionex corp. Sunnyvale, CA USA) equipped with a Hypersil GOLD column (150 mm × 2.1 mm; 3 µm, Thermo Fisher Scientific. Waltham, MA, USA). The chromatography was performed at 0.4 mL/min at 40 °C. The eluent system comprised a two-eluent system with MilliQ water (A) and acetonitrile (B). In addition, 0.01% formic acid was present at all times. The elution profile is given in % B. Linear gradient from 20 to 90% B in 0.5 min, isocratic 90% B for 2 min, followed by re-equilibration at 20% B for 3.5 min. The electrospray was operated in negative mode with UltraScan mode and a scan range from 100 to 1000 m/z, smart parameter setting of 190 m/z, capillary voltage at 4.5 kV, endplate off-set 0.5 kV, nebulizer pressure at 3.0 bar, dry gas flow at 12.0 L/min, and dry gas temperature at 280 °C. CID fragmentation of deprotonated ions was performed by Multiple Reaction Monitoring using SmartFrag enhanced amplitude ramping from 80 to 120%, fragmentation time 20 ms. Quantification was performed in Compass QuantAnalysis 2.2 (Bruker Daltonics, Bremen Germany) using trans-ferulic acid as external standard and adjusted according to the internal standard as described previously [38].

### Matrix-assisted laser desorption/ionization-time of flight (MALDI-TOF) characterization of oligosaccharides

One μL sample (arabino-, fructo-, or galacto-oligosaccharides, 10 mg/mL oligosaccharide dissolved in MilliQ water) and 1 μL of 10 mg/mL di-hydroxy benzoic acid matrix in 50% acetonitrile was applied to a MTP 384 target plate ground steel BC (Bruker Daltonics GmbH, Bremen, Germany), mixed on the target and dried under a stream of air. The samples were analyzed with an Ultraflex MALDI-TOF/TOF instrument (Bruker Daltonics GmbH, Bremen, Germany). The instrument was operated in positive acquisition mode and controlled by the FlexControl 3.3 software package. All spectra were obtained in reflector mode with the least required laser intensity and pulsed ion extraction of 40 ns. The acquisition range used was from m/z 300 to 3000.

### Piglet colon content used as inoculum for in vitro fermentations

The experiment was carried out with respect to ethical procedures for animal experimentation and with approval from the Danish Animal Experimentation Inspectorate, license number (2016-15-0201-01018). Colons from five freshly sacrificed weaned piglets aged 32 days were dissected and colon content removed for 16S rRNA gene amplicon sequencing-based analysis and as inoculum for in vitro fermentations. The pigs were from a commercial piggery with 1600 sows (Danish Landrace x Danish Yorkshire, Danbred sows) and were a subsample of a larger growth trial involving 284 piglets from 64 different sows over a period of 5 weeks [39].

Colons were dissected, and the colon content mixed 1:1 with a 20% glycerol and PBS mix and homogenized in a stomacher (Seward, UK) for 2 min at normal speed, aliquoted, frozen, and stored at − 80 °C until further use in in vitro fermentations. Fermentations (n = 4 for each fermentation) were performed with colon content pooled from 5 healthy, freshly weaned piglets.

### In vitro fermentations

The in vitro fermentations were carried out using the CoMiniGut-system as described previously [34], but with fermentation parameters adjusted to simulate the piglet intestine. The temperature was hence adjusted to simulate a body temperature of 38 °C [40, 41] and the passage through the piglet intestine was simulated through the controlled increment of pH from 6 to 6.5 pH throughout the 24 h of in vitro fermentation of the piglet GM and substrate [42]. Details on assembly of fermentation chambers, inoculation (1% final concentration of colon content), maintenance of anaerobic fermentation conditions and dynamic pH-control during the fermentations has been lined out previously [34]. Basal colon media previously used for in vitro screening [43–45] was adjusted to simulate the porcine gastro-intestinal tract by supplementing it with 1 g/L porcine mucin (Sigma-Aldrich) and reducing the amounts of yeast extract and peptone water (0.5 g/L each compared to standard basal medium). Each CoMiniGut reactor was hence filled with 4.5 ml basal medium (0.5 g/l bile salts, 1.5 g/l peptone water, 1.0 g/l yeast extract (Oxoid), 1.0 g/l porcine mucin (Sigma-Aldrich), 0.1 g/l NaCl, 0.04 g/l K_2_HPO_4_, 0.04 g/l KH_2_PO_4_ (Merck), 0.01 g/l MgSO_4_ 7H_2_O, 0.01 g/l CaCl_2_ 6H_2_O, 2 g/l NaHCO_3_, Hemin 0.002 g/l, Vitamin K1 10 µl, Tween-80 2 ml (Sigma-Aldrich), 0.5 g/l L-Cysteine HCl (Calbiochem, San Diego, CA, USA). The media was supplemented with 0.5 ml of the substrates at concentrations as listed in Table 1. For no substrate controls the media was added 0.5 ml sterile MilliQ-water. All in vitro fermentations were carried out in quadroplicate.

### Short chain fatty acids (SCFA) extraction and analysis

SCFA were extracted from 1 ml fecal slurry collected at fermentation endpoint (24 h) as reported previously [34]. In brief, two ml of 0.3 M oxalic acid was added to the sample and vortexed for 1 min, followed by centrifugation at 2800 g for 15 min. A 1 µl of filtered clear supernatant was injected into a GC–MS using a split/split-less inlet operated at 285 °C and using a 2:1 split ratio. The GC oven program was as follows: initial temperature 100 °C, equilibration time 1.0 min, heat up to 120 °C at the rate of 10 °C/min, hold for 5 min, then heat at the rate of 40 °C/min until 230 °C and hold for 2 min. Mass spectra were recorded in Selected Ion Monitoring (SIM) mode, and the following m/z ions were detected: 41, 43, 45, 57, 60, 73, 74, 84. The MS detector was switched off during the 1 min solvent delay time. The transfer line, ion source, and quadrupole MS temperatures were set to 230, 230, and 150 °C, respectively. For each type of determined SCFA, including total SCFA, *t* test was applied for pairwise comparison between substrate groups, and the original *p* values were corrected with the Benjamini & Hochberg method.

### Sampling and DNA extraction

One ml of fecal slurry (inoculum) (see above) and sample material after at 24 h of fermentation were pelleted via centrifugation at 13.000 g for 10 min and gDNA was extracted from the pellet using the Power Soil Kit protocol (MoBio Laboratories, Carlsbad, CA, USA). The FastPrep bead-beating step was performed in 3 cycles of 15 s each at a speed of 6.5 M/s in a FastPrep-24TM Homogenizer (MP). DNA quantity and quality were measured using a NanoDrop 1000 (Thermo Scientific, Waltham, MA, USA).

### 16S rRNA gene amplicon library preparation and bioinformatics

The microbiota composition of in vitro fermentation samples was profiled using tag-encoded V3-region 16S rRNA gene NextSeq-based (Illumina, CA, USA) high throughput sequencing as previously described [46]. Paired raw reads were filtered and trimmed for ASV (amplicon sequence variant) inference based on DADA2 (v1.12.1) algorithm [47] after removing amplification primers with cutadapt (v2.4). Denoised reads were merged to construct a non-redundant ASV catalogue after removing chimera in the consensus mode of DADA2. ASV sequence and abundance matrix were imported to QIIME2 (2019.07) [48] for taxonomic classification and phylogenetic tree construction. Naïve Baysian classifier was trained with q2-feature-classifier on the exact amplified area using Green Gene 99% OTU sequences. The minimum frequency of ASVs was set as 0.1% of the mean sample depth to avoid bleeding-through between sequencing runs. ASVs unclassified at phylum level were removed. A rooted phylogenetic tree of remaining ASVs was constructed with fasttree in QIIME 2. Cross-contaminated ASVs during laboratory preparation were identified independently in different batches using default prevalence mode of decontam [49]. One ASV, classified as *Escherichia coli*, was removed considering high relative abundance (> 50%) in all the negative samples. Following the recently published re-organised taxonomy of the former *Lactobacillus* genus [50], basonym of *Lactobacillus* spp. was substituted by new taxonomic naming with the online tool (http://lactobacillus.ualberta.ca). In the following, the term “lactobacilli” refer to taxa previously belonging to the *Lactobacillus* genus [50].

The final abundance matrix, taxonomy, sample data, and tree file were imported to R through phyloseq [51] for data analysis. Experimental replicates with abnormal O_2_ indicator or skewed pH-profile during the CoMiniGut-run were discarded. The remaining samples were rarefied to an even library depth of 14,000 reads using “rarefy_even_depth” in phyloseq to calculate Shannon index (alpha diversity) and weighted and unweighted Unifrac distance metrics (beta diversity). Pairwise Wilcoxon test and PERMANOVA were performed to determine statistically significant differences in alpha and beta diversity metrics, respectively. The raw ASV table was summarized at the lowest classified level. Responding taxonomic features were extracted through comparison between media control and respective experiment group using DESeq2 [52] with a preset adjusted *p*-value of 0.05. The responding taxa were integrated for heatmap visualization using heatmap.2 in R package gplots. Pearson’s correlation analysis (Rhea package) [53] was conducted between centered log-ratio transformed relative abundances at species level and SCFA data.

## Results

Twenty different substrates (including 16 pectin derived substrates) were tested for their effect on the piglet colon GM during 24 h of in vitro incubation. For comparison, the GM modulating properties of known prebiotics, namely FOS, GOS, inulin and the polysaccharide arabinogalactan, were also included in the study. Fermentations without addition of substrate were included as blank controls.

### The influence of pectin type on the in vitro simulated colon microbiome

**Fig. 1:**
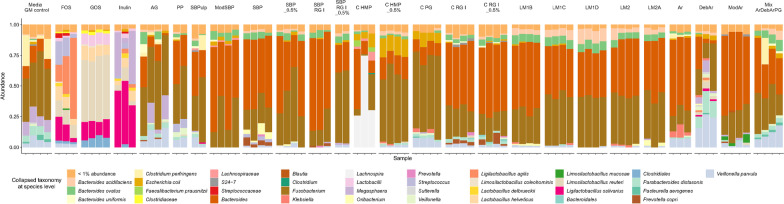
Piglet gut microbiota composition (summarized on species level) as determined by 16S rRNA gene (V3-region) amplicon sequencing after in vitro simulated colon fermentation of different substrates as indicated. Only taxa with average relative abundance > 1% shown. All fermentations were carried out in quadruplicate unless otherwise stated. The additive dose is 1% except for groups with specific description of using 0.5% additive dose. Media-GM control, no added pectin saccharides, *FOS* fructooligosaccharides derived from chicory (n = 3), *GOS* galactooligosaccharides derived from milk, *Inulin* inulin derived from chicory (n = 3), *AG* arabinogalactan, *PP* pea pectin (n = 2), *SBPulp* pulp from sugar beet (n = 2), *ModSBP* modified sugar beet pectin, *SBP* sugar beet pectin, *SBP_0.5%* same, but 0.5% substrate; SBP_RG-I (n = 3) and SBP-RG-I_0.5 (n = 2), sugar beet pectin derived rhamnogalacturonan I, C-HMP and C-HMP_0.5, citrus derived high methoxyl pectin (n = 3) 1% substrate, C-*PG* citrus derived polygalacturonic acid (constituting pectins through methyl esterification), *C-RG-I* citrus derived rhamnogalacturonan I, *C-RG-I_0.5%* LM1B, low methoxyl pectin, random esterification pattern, *LM1C* low methoxyl pectin, random esterification pattern, *LM1D* low methoxyl pectin, random esterification pattern, *LM2* low methoxyl pectin, blocky esterification pattern, *LM2A* low methoxyl pectin, blocky esterification pattern (n = 3), *Ar* arabinans derived from sugar beet pectin, *DebAr* debranched arabinans derived from sugar beet pectin (n = 3), *ModAr* modified arabinans, *MixArDebArPG* 1:1:1 substrate mixture of polygalacturonic acid, arabinans and debranched arabinans. See Table 1 for details on degree of esterification and acetylation for the different substrates

After 24 h of in vitro simulated passage of the piglet colon FOS, GOS and inulin led to an increase of *Firmicutes* dominated by *Ligilactobacillus* (former *Lactobacillus*) *agilis*, *Limosilactobacillus* (former *Lactobacillus*) *reuteri* and *Ligilactobacillus* (former *Lactobacillus*) *salivarius*. In contrast, arabinogalactan led to a GM dominated by *Fusobacterium* and *Bacteroides* (Fig. 1; Additional file 1: Fig. S1). Pectin and pectin-derived saccharides in most cases resulted in GM dominated by *Bacteroides* with substrate molecular structure-specific differences depending on the source of pectin (Fig. 1). The pectin-derived substrates were classified into four categories by pectin source and derived polysaccharides components; degree of esterification and pattern, the dose of pectin substrate (1% and 0.5%), and pectin derived oligosaccharides. Except for C-RG-I, citrus-derived substrates (C-HMP, C-PG) led to reduced relative abundance of *Bacteriodes* and promoted the growth of *Escherichia coli* relative to sugar beet-derived pectins (Fig. 1). C-HMP also promoted the growth of *Lachnospira* and *Faecalibacterium prausnitzii* relative to all other substrates (Additional file 1: Fig. S2). Interestingly, an ASV identified as *Clostridium perfringens* was present at a relative abundance of 3.7% in the control and 5.9% in the pulp fermentations, but only 0.01–0.02% in the  SBP and SBP-RG-I fermentations (Fig. 1).

**Fig. 2:**
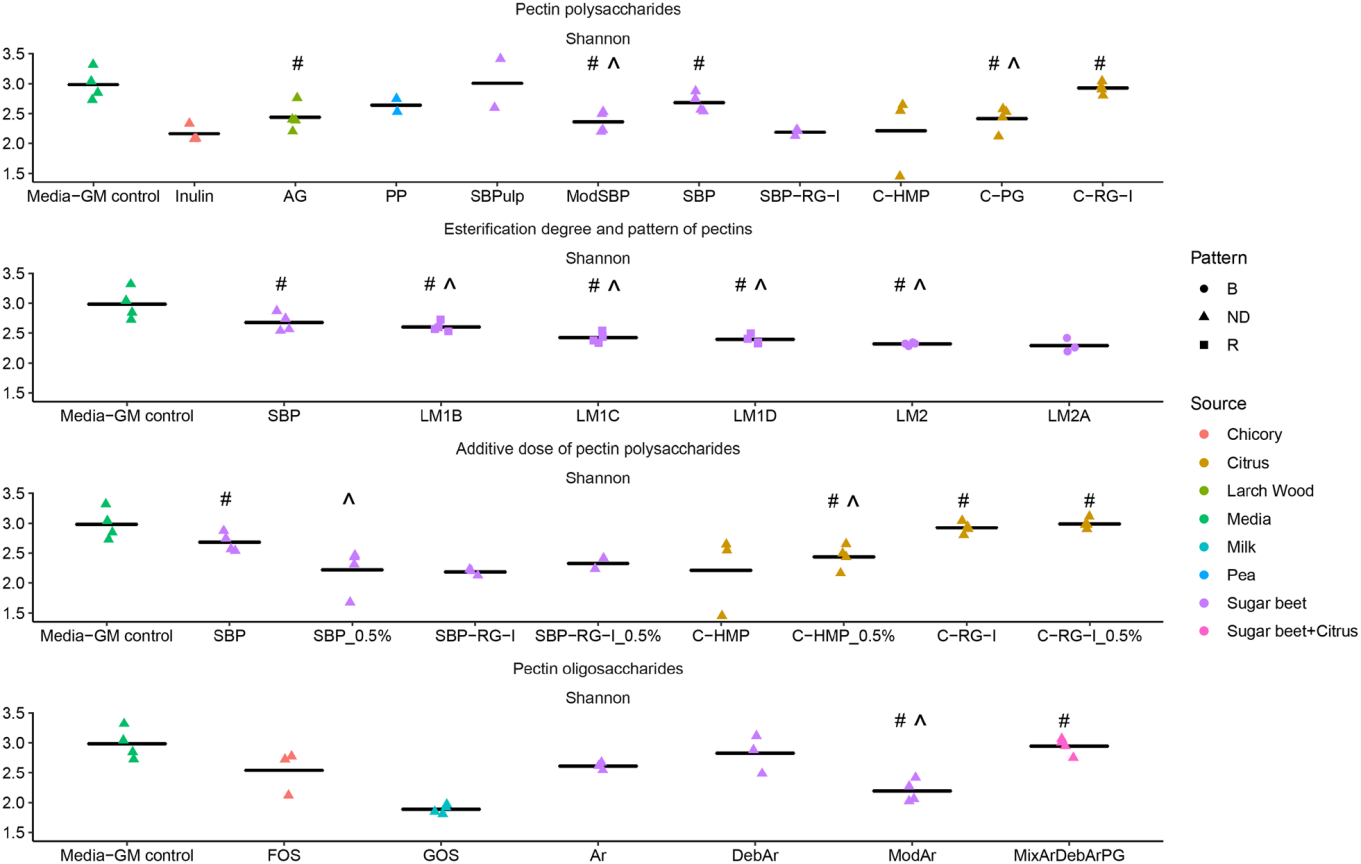
The effect of substrate on Shannon diversity index of in vitro simulated piglet gut microbiota as determined by 16S rRNA gene (V3-region) amplicon sequencing. The additive dose is 1% except for groups with specific description of using 0.5% dose. The data shown are averages of 4 replicates, unless otherwise stated. **a** Polysaccharides sourced from pectins with inulin (n = 3) and AG (arabinogalactan) as prebiotic controls. *PP* pea pectin (n = 2), *SBPulp* pulp from sugar beet (n = 2), *ModSBP* modified sugar beet pectin, *SBP* sugar beet pectin, *SBP_RG-I* sugar beet pectin derived rhamnogalacturonan I (n = 3), *C-HMP* citrus derived high methoxyl pectin(n = 3 for 1% dose), *C-PG* citrus-derived polygalacturonic acid (constituting pectins through methyl esterification), *C-RG-I* citrus derived rhamnogalacturonan I; **b** sugar beet pectins with different degrees and patterns of esterification. *SBP* sugar beet pectin, *LM1B* low methoxyl pectin, random esterification pattern, *LM1C* low methoxyl pectin, random esterification pattern, *LM1D* low methoxyl pectin, random esterification pattern, *LM2* low methoxyl pectin, blocky esterification pattern, *LM2A* low methoxyl pectin, blocky esterification pattern (n = 3); **c** different pectin polysaccharides at additive dose of 1% and 0.5%. **d** Oligossaccharides derived from pectins with FOS (fructooligosaccharides, n = 3) and GOS (galactooligosaccharides) as prebiotic controls. *AR* arabinans derived from sugar beet pectin (n = 3), *DebAr* debranched arabinans derived from sugar beet pectin (n = 3), *ModAr* modified arabinans, *MixArDebArPG* 1:1:1 substrate mixture of polygalacturonic acid, arabinans and debranched arabinans. In all cases compared against FOS, GOS and inulin (n = 3). *Significantly different from FOS; #significantly different from GOS and ¤significantly different from inulin; ^significantly different from Media-GM control, the significant marks are assigned if P values < 0.05. See Table 1 for details on degree of esterification and acetylation for the different substrates

**Fig. 3:**
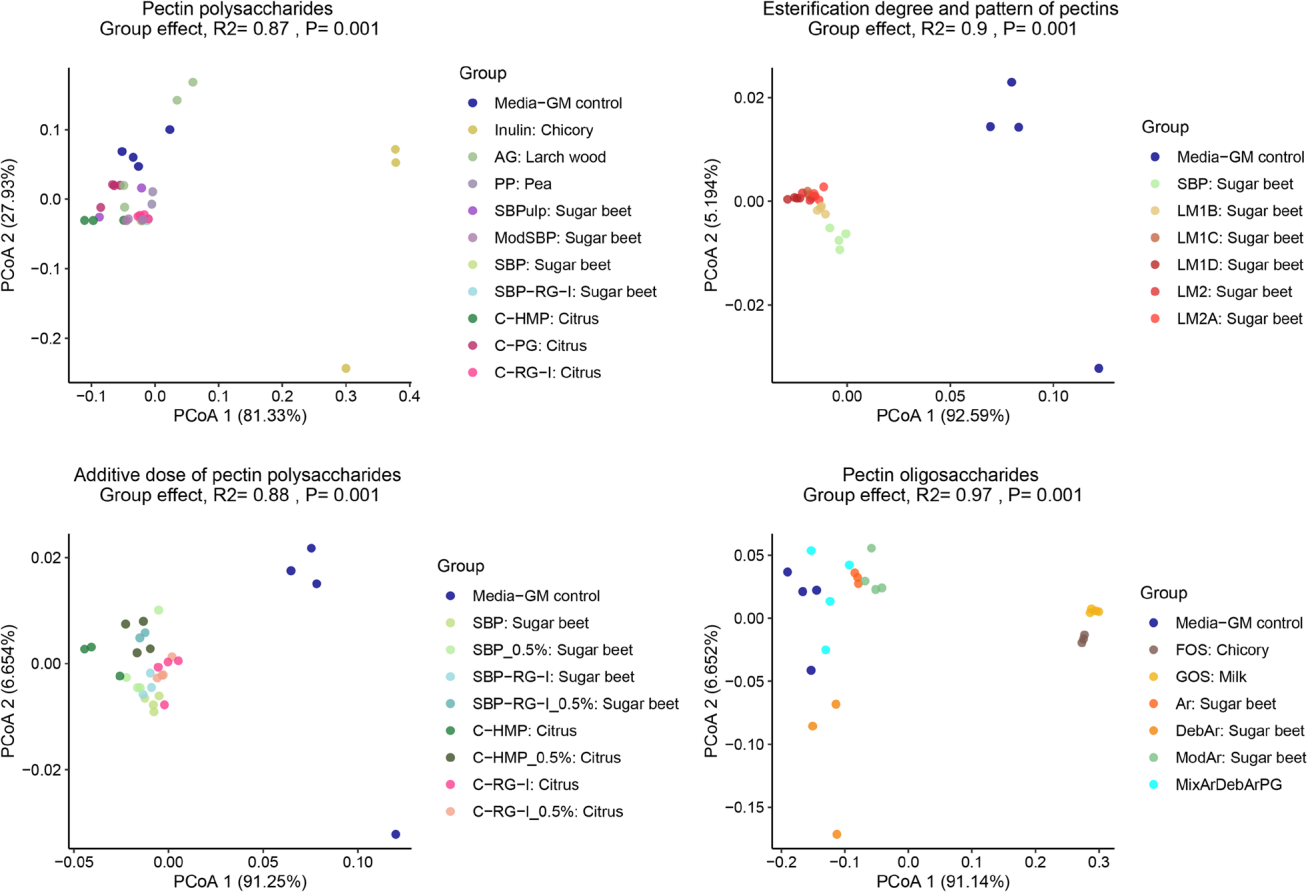
The effect of different substrates on in vitro simulated piglet gut microbiota as determined by 16S rRNA gene (V3-region) amplicon sequencing (Weighted Unifrac distance metrics). All fermentations were carried out in quadruplicate (with exceptions as stated in Fig. 1). **a** Polysaccharides sourced from pectins with inulin and AG (arabinogalactan) as prebiotic controls. **b** Sugar beet pectins with different degrees and patterns of esterification. **c** Different pectin polysaccharides at additive dose of 1% and 0.5%. **d** Oligossaccharides derived from pectins with FOS (fructooligosaccharides) and GOS (galactooligosaccharides) as prebiotic controls. See Table 1 substrate abbreviations and details on degree of esterification and acetylation for the different substrates

**Fig. 4:**
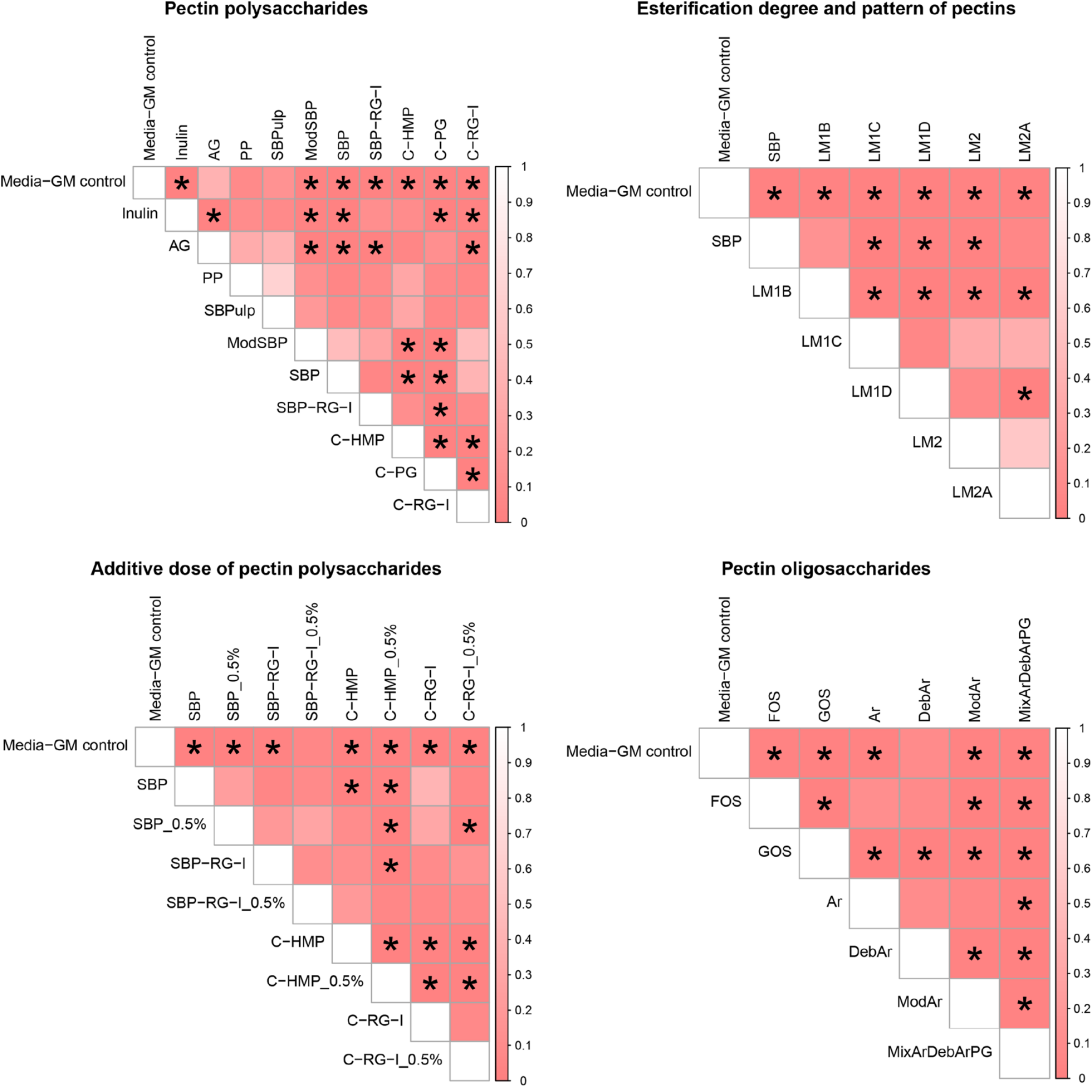
Pairwise PERMANOVA tests on weighted Unifrac distance metrics visualized as heatmaps. Substrates were grouped to allow targeted comparisons: Polysaccharides sourced from pectins with inulin and AG (arabinogalactan) as prebiotic controls (**a**). Sugar beet pectins with different degrees and patterns of esterification (**b**). Different pectin polysaccharides at additive dose of 1% and 0.5% (**c**). Oligossaccharides derived from pectins with FOS (fructooligosaccharides) and GOS (galactooligosaccharides) as prebiotic controls (**d**). *P < 0.05. All fermentations were carried out in quadruplicate (with exceptions as stated in Fig. 1)

Shannon diversity indexes are shown in Fig. 2, while weighted Unifrac distance-based PCoA plots are visualized in Fig. 3. Statistically significant pairwise comparisions for Shannon diversity and PERMANOVA (Weighted Unifrac) can be found in Additional file 2: Table S1 and Fig. 4. Moreover, to discern the effect of added substrate on the GM community structure, we conducted DESseq2 analysis to determine the corresponding taxa of each substrate against the media GM control (visualized in Additional file 1: Fig. S2).

Overall, in vitro fermentation for 24 h with pectin-derived substrates resulted in decreased Shannon diversity index relative to the media control, suggesting that these saccharides favored the growth of specific bacteria leading to their dominance. SBP-pulp, probably attributed to the low bioavailability of the SBP-pulp particles, resulted in a Shannon diversity index not significantly different from the media GM control. GOS provided favorable conditions for substantial proliferation of *Limosilactobacillus* (former *Lactobacillus*) *reuteri* (Fig. 1), resulting in the lowest Shannon diversity among all substrate groups.

The investigated substrates influenced overall community structure (as determined by weighted Unifrac distance metrics) differently depending on the specific substrate component, the degree of esterification, and the substrate concentration (Fig. 3). Pairwise PERMANOVA was conducted to discern statistically significant differences between substrates (Fig. 4). Of the pectin-substrates only pea pectin and SBPulp did not lead to a community structure statistically different from the microbiota of the no substrate control. We did not observe statistically different community structures between sugar beet-derived polysaccharide substrates such as SBP, ModSBP and SBP-RG-I, and PP (Fig. 4). However, the GM profiles promoted by sugar beet derived substrates differed significantly from the profiles promoted by the common prebiotics inulin, FOS and GOS, and showed potential to increase the relative abundance of *Bacteroides* members relative to the blank control (Additional file 1: Fig. S2).

The degree of esterification (DE) varies between different pectin molecules and can be modified during pectin substrate production. Here, we investigated the effect of different DE and different esterification patterns, namely random vs. blocky, of sugar beet pectin substrates on their fermentation by weaner GM. While the Shannon diversity of both SBP and most LM-based substrates was higher than GOS, the different estericiation patterns did not lead to significant differences in Shannon diversity. At the community level, SBP pectins of different esterification degrees and patterns led to distinct communities different from media control but also clustering according to the substrate in the majority of cases (Figs. 3 and 4). The LM SBP pectin substrates with random as well as blocky esterification pattern promoted a trend towards a higher relative abundance of *Bacteroides ovatus* and *Bacteroides acidifaciens* within the GM community (Fig. 1).

For substrates SBP, SBP-RG-I, C-HMP and C-RG-I the effect of substrate dose (0.5% vs 1%) was investigated. Dose variation did not influence Shannon diversity index to any larger extent (Fig. 2; Additional file 2: Table S1). As determined from weighted Unifrac metrics only for C-HMP did the investigated doses influence community structure differently (Fig. 4). Interestingly, C-HMP promoted growth of *Lachnospira* at 1% w/v and *Fusobacterium* at 0.5% after 24 h of fermentation (Fig. 1).

We investigated the effect of FOS, GOS and SBP derived arabinan oligosaccharides of different properties (branched, debranched, modified) to evaluate their impact on the weaner GM in vitro. FOS and GOS, promoted a very distinct microbial community in vitro, which differed significantly from the GM stimulated by SB-derived oligosaccharide substrates (Figs. 3 and 4). FOS led to a community dominated by *Ligilactobacillus* (former *Lactobacillus*) *agilis, Ligilactobacillus* (former *Lactobacillus*) *salivarius*, *Megasphaera* and a member of *Streptococcus*. GOS supported the growth of various lactobacilli such as *Limosilactobacillus* (former *Lactobacillus*) *reuteri, Lig. salivarius, Lactobacillus helveticus* and unclassified *Lactobacillus* (Fig. 1). Similar to inulin fermentations both oligosaccharides supported a GM community with *Fusobacterium* and *Bacteriodes* not detected, whereas these two genera generally dominated control and sugar beet substrate fermentations. Interestingly, while modified arabinans promoted the growth of *Limosilactobacillus* (former *Lactobacillus*) *mucosae*, the remaining GM community resembled the effects of the SBP supplemented fermentations (Fig. 1). Debranching of sugar beet derived arabinan molecular structures had a signficant impact on the modulation of the GM community in vitro. Debranched arabinans selectively promoted the growth of *Veillonella parvula*, *Bifidobacterium* and some lactobacilli such as *Lim. reuteri* and *Lig. salivarius* (Additional file 1: Fig. S2).

Polygalacturonic acid from citrus in a 1:1:1 mixture with debranched and branched arabinans (MixArDebrArPG) was tested to investigate the effect of a mixed oligosaccharide composition on the weaner GM in vitro. This mix led to a higher Shannon diversity index than observed for the Ar and PG alone, favoring the growth of *Bifidobacterium*, *Veillonella parvula* and *Bacteriodes* like *Bacteroides ovatus*, *Bacteroides acidifaciens*, and *Bacteroides uniformis* (Additional file 1: Fig. S2).

### The influence of pectin type on SCFA production during in vitro simulated colon fermentations

**Fig. 5:**
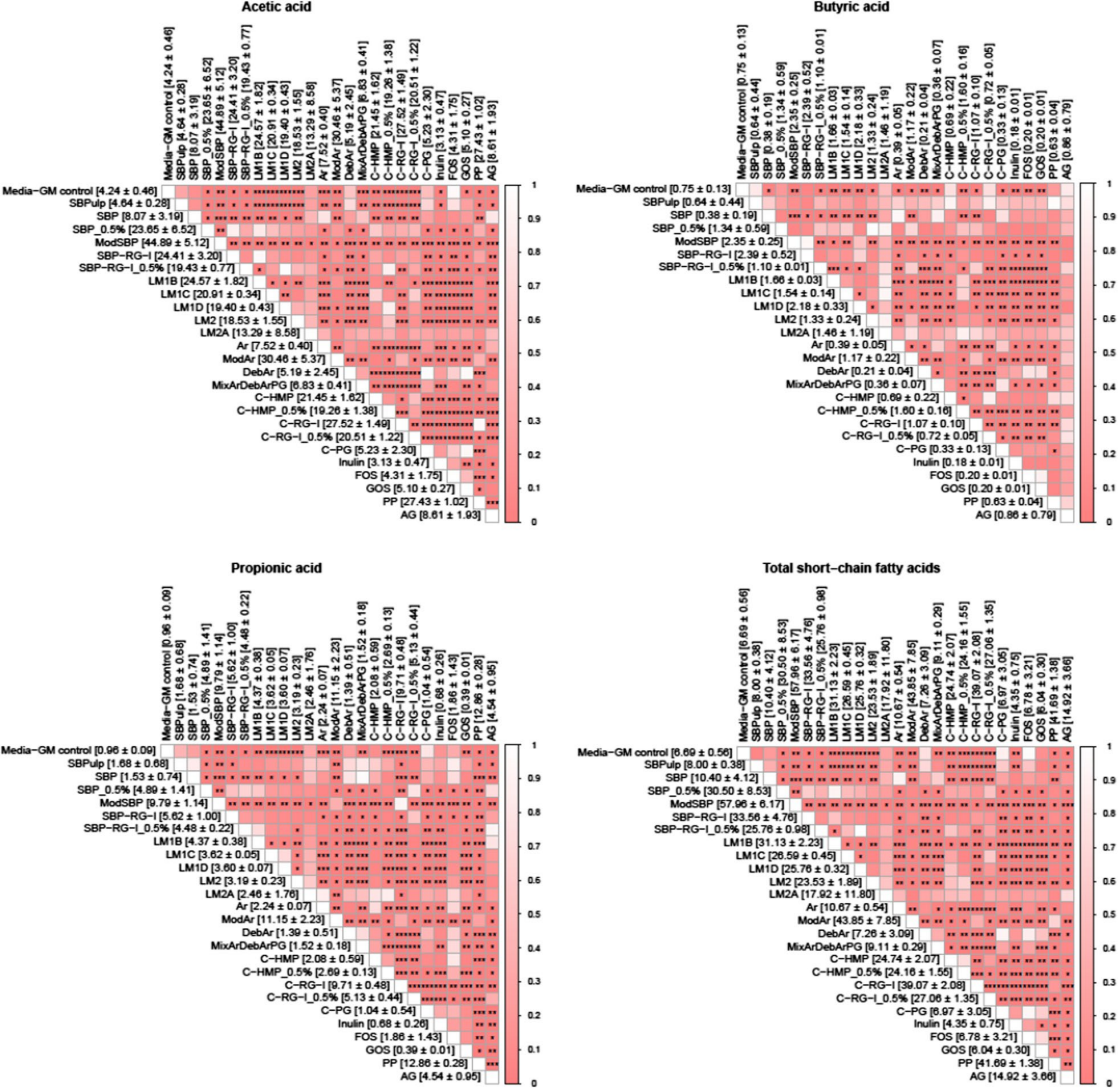
Pairwise comparison of the amount of the short chain fatty acid (SCFA) produced from different substrates as determined by *t*-test with FDR correction. The comparisons are carried out for acetic acid, propionic acid, butyric acid as well as total SCFA. All fermentations were carried out in quadruplicate (with exceptions as stated in Fig. 1). The symbols *, **, ***represent adjusted P < 0.05, 0.01 and 0.001, respectively. The colour depth of each cell represents the adjusted P value. The values in the square brackets indicate the mean and standard deviation of the individual SCFA. Substrate codes as in Table 1. MixArDebArPG indicate mixed fermentation 1:1:1 (each 0.33% w/v) of arabinan, debranched arabinan, and polygalacturonic acid

The SCFA profiles were strongly influenced by the different pectin sources and their influence on the microbiota composition, as seen from Fig. 5 and the hierarchically clustered heatmap (Additional file 1: Fig. S4). Interestingly, sugar beet derived substrates yielded higher total SCFA (total SCFA: Mod-SBP 57.97 mM, SBP-RB-I 33.50 mM, SBP 10.40 mM) than the known prebiotics inulin (total SCFA 4.35 mM), FOS (6.78 mM), and GOS (6.04 mM), with Mod-SBP resulting in the highest values with acetate and propionate being produced in high amounts (Fig. 5). Overall, most of the tested pectin-derived compouns yielded significantly higher SCFA amounts that inulin, FOS and GOS, respectively (Fig. 5). Further, it was found that low DE pectins led to 2–3 times higher SCFA concentrations relative to SBP pectins (Fig. 5). Also, modified arabinans yielded high amounts of SCFA (total SCFA 43.85 mM), whereas SBP derived arabinans (9.19 mM) and debranched arabinans (7.26 mM) yielded relatively low amounts of SCFA (Fig. 5).

High colonic levels of butyrate is generally positively linked to beneficial health effects. We found that SBP-RG-I yielded the highest amount of butyrate (2.51 mM) when fermented at 1% (w/v) compared to 1.10 mM when fermented at 0.5% (w/v). Butyrate production was influenced by DE. All LM pectins yielding higher butyrate levels than SBP pectin, and LM-1D facilitated the highest butyrate production amongst the LM pectin substrates indicating that a lower DE promotes butyrate production (Fig. 5). The fermentation of pea pectin yielded the highest amount of propionate (12.53 mM), followed by fermentations supplemented with arabinans of mixed structure, ModSBP and C-RG-I. All LM pectins also yielded more propionate than SBP, indicating that different molecular structures impact their bioavailability and metabolization by the weaner GM and the metabolites produced.

Sugar beet substrates yielded the highest amounts of acetic acid, with ModSBP and (44.89 mM), ModAr (30.46 mM), and pea pectin (27 mM) being the substrates that promoted acetic acid production the most (Fig. 5). The oligosaccharides were on the other hand, relatively poor substrates for acetic acid production (Fig. 5).

### Relationship between bacterial taxa and SCFA production

**Fig. 6:**
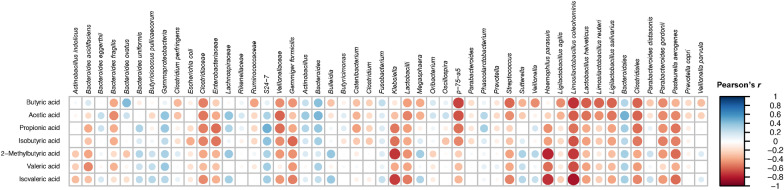
Correlation (Pearson) between microbial taxa relative abundance and short- and branched- chain fatty acid concentrations. The minimal prevalence (among the sequenced samples) of one given microbial taxa is set to 30%

Through Pearson’s correlation between bacterial taxa and SCFA profiles, we found 109 significant pairs after multiple testing corrections (Additional file 3: Table S2). Interestingly, most lactobacilli were negatively correlated with produced SCFAs, while *Bacteriodales* members positively correlated with SCFA concentrations (Fig. 6). The prebiotics FOS, GOS, and inulin lead to distinct microbial communities occupied by lactobacilli. Still, the SCFA-producing ability of these communities was in general lower than that of the communities grown with added pectin-derived substrates.

## Discussion

Only little is known about the structure–function relationship of sugar beet-derived pectic molecules on GM and GM function. Existing studies generally provide little information about the pectin used, the source, and its molecular structure. These differences in study design hinder a comprehensive understanding of the potential of pectin to impact pig nutrition and health, as recently reviewed [33]. Pectin properties can differ in the source and structural features such as fine branching, DE, degree of acetylation, and molecular weight. In this study, we aimed at elucidating the structure to function relationship of pectin on the piglet weaner GM, revealing especially the effect of different sugar beet pectin components on GM and SCFA-production.

Sugar beet-derived substrates such as SBP and SBP-RG-I both increased the relative abundance of *Bacteroides.* In healthy mammals, 20–80% of the intestinal microbiota belong to the *Bacteroidetes* phylum. They play important roles not only degrading otherwise undigestable carbohydrates but also being essential for immune system developement and function [54, 55]. Interestingly, we found that the pectin structure and DE have an impact on the specific *Bacteroides* genera promoted within the weaner GM in vitro. Both the SBP pectin substrates with blocky and with random esterification patterns overall increased the relative abundance of *Bacteroides ovatus* and *Bacteroides acidifaciens*. Our study is one of few to investigate the effect of the DE and the effect of a specific esterification pattern (blocky and random) on the GM. The finding that pectin substrates with relatively low DE lead to a GM with relatively high diversity (Fig. 2) is interesting as a higher microbial diversity in several studies has been associated with better resistance towards perturbations and thus resilience [56, 57].

Interestingly, the SBP derived substrates (SBP, SBP-RG-I) and inulin reduced some ASVs of clostridia, most notably an ASV of *Clostridium perfringens*, which was present at a relative abundance of 3.7% in the control and 5.9% in the pulp fermentations, but only 0.01–0.02% in the SBP and SBP-RG-I fermentations. *Clostridium perfringens* is associated with various GIT disorders in weaned piglets and neonates; for instance, *Cl. perfringens* type C is generally considered to be the primary cause of necrotic enteritis in piglets aged 0–2 weeks [58, 59], and type A has been linked to enteric disease in suckling and feeding pigs with mild necrotic enterocolitis and villous atrophy [60–62]. Keeping the presence of *Cl. perfringens* as low as possible in the production units is thus of high interest.

Citrus-derived pectins promoted other potentially beneficial bacteria, such as C-HMP which promoted *F. prausnitzii* relative abundance. *Faecalibacterium prausnitzii* is an important butyrate producer in the gut and has been observed to suppress inflammation by blocking the NF-κB pathway and inducing regulatory T cells [63, 64]. *Faecalibacterium. prausnitzii* has previously been shown to utilize apple pectin and galacturonide oligosaccharides from sugar beet in in vitro models [65] and taken together these data indicate that several types of pectin have the potential to stimulate *F. prausnitzii*.

Other *Firmicutes* members like *Lim. reuteri* were thriving on citrus derived pectins such as RG-I, while modified arabinans promoted the growth of *Lim. mucosae*. *Limosilactobacillus reuteri* produces a range of antimicrobial compounds, and certain strains have been associated with reduced incidence of necrotizing enterocolitis in preterm piglets [66] and *L. mucosae* are have been investigated as putative probiotics due to their metabolic capabilities and ability to colonize host mucosal niches [67]. In line with this, a recent study by Wilkowska et al. reported that sugar-beet pulp after combined pre-treatment by yeast cultivation and pectin hydrolysis resulted in a hydrolysate with prebiotic effect stimulating the growth of lactobacilli and bifidobacteria, while pathogens such as *Staphyloccoccus aureus* and *Salmonella* grow poorly [68].

In nature, porcine milk oligosaccharides display a high diversity of molecular structures and a mix of different oligosaccharide types. For this reason, we also explored the behaviour of substrate mixes in vitro. Polygalacturonic acid from citrus in a 1:1:1 mixture with debranched and branched arabinans was tested to investigate the effect of a mixed oligosaccharide composition on the weaner GM in vitro. The highest Shannon index was detected for the substrate mix MixArDebrArPG, indicating that mixing of substrates might have potentially beneficial effects on GM structure and the ability to resist perturbations. FOS and GOS are common oligosaccharides, that are used as additives in infant formula and can also be used as additives in weaner feed. Of note, we have found that inulin, FOS and GOS were inferior to most tested pectins with respect to total amounts of SCFA produced. Uerlings et al. investigated SCFA-production during in vitro simulated colon digestion using piglet feaces as inoculum and pectin from a range of sources (sugar beet pulp, citrus pulp, purified pectin from citrus etc.) as substrate and overall found, that inulin as substrate resulted in similar or slightly higher total SCFA values than the pectic substances [69]. This is contrary to the present study, where e.g. ModSBP and LM-pectins resulted in significantly higher SCFA-levels than inulin (Fig. 5). A likely explanation of the discrepancy between the two studies, is that the tested pectin substances are not the same and hence not directly comparable. It can speculated, that the exposure to sow fecal matter in the period up to weaning (after which the colon material used as inoculum in the present study) exposes the piglets to bacteria with a higher capacity for SCFA-production from pectic substances (from the feed), than oligosaccharides, but the exact reason remains to be elucidated. Another reason could be, that FOS and GOS is partly fermented into lactate. The piglet GM is rather high in lactobacilli producing lactate, but unfortunately lactate was not measured, and hence this hypothesis cannot be confirmed.

Using human fecal matter as inoculum, the degree of methylation (DM) has previously been shown to influence the amount of propionate produced during in vitro colon fermentations, with high methylated pections generally leading to more propionate, than low methylated pectins [70]. We also demonstrate that pectin structure has a strong effect on SCFA production (Fig. 5). Due to the larger number of structures modified from the same substrate investigated in the present study, our data show that the relationship between SCFA-production and pectin structure is influenced by more factors than DM. It is seen that the SBP LM pectin with the highest DE (LM1B) leads significantly more acetate and propionate than SBP LM pectins with lower DE. Further, and perhaps not so surprising, the partly insoluble SBPulp and PG resulted in low SCFA amounts, while ModAr, which compositionally is close to Ar and DebAr resulted in significantly higher concentrations of all 3 tested SCFAs than the latter two substrates. Size (in kDa) does not seem to systematically influence SCFA production from the investigated substates.

There is limited knowledge on the dose–response of prebiotics in piglets. Here, we demonstrate that dose in some cases does matter and that different doses can easily be tested in vitro to assess the effect on GM and its SCFA production. Interestingly, C-HMP promoted growth of *Lachnospiraceae* at 1%, but *Fusobacterium* at 0.5% (Fig. 1). A possible explanation could be, that at 0.5% the microbiome run out of fermentable pectins before 24 h of fermentation, which might give *Fusobacterium* a competitive advantage. In vivo testing of feed components is tedious and costly. In vitro derived data can help qualify in vivo trials ensuring that the most promising candidates are investigated which in turn will reduce the cost of developing new feed additives.

## Conclusion

In conclusion, we demonstrate how pectins derived from different raw materials and with different molecular structures (e.g. DP, DE, DA) have different effects on weaner piglet gut microbiome composition and function (SCFA-production) in vitro. With respect to SCFA production, the majority of the pectins performed better or at least as good as the already known and widely used prebiotics inulin, FOS and GOS. The findings in the present study thus opens new possibilities for rapidly designing novel pectin-containing piglet feed mixtures with targeted effects on GM composition and function for in vivo testing.

## Supplementary Information

**Additional file 1. Fig. S1**: Piglet gut microbiota composition (phylum level) as determined by 16S rRNA gene (V3-region) amplicon sequencing after in vitro simulated colon fermentation of different substrates. All fermentations were carried out in quadruplicate (with exceptions as stated in Fig. 1). See Table 1 for substrate abbreviations and details on degree of esterification and acetylation for the different substrates. **Fig. S2** Representative microbial taxa of different substrate group relative to media control were plotted in one integrated heatmap (determined by DESeq2 on the summarized lowest classified levels). All fermentations were carried out in quadruplicate (with exceptions as stated in Fig. 1). See Table 1 for substrate abbreviations and details on degree of esterification and acetylation for the different substrates (will be uploaded as high-resolution). **Fig. S3**. Pairwise comparison of the amount of the branched chain fatty acid (BCFA) produced from different substrates as determined by t test with FDR correction. All fermentations were carried out in quadruplicate (with exceptions as stated in Fig. 1). The symbols *, **, *** represent adjusted P < 0.05, 0.01 and 0.001, respectively. The colour depth of each cell represents the adjusted P value. The values in the square brackets indicate the mean and standard deviation of the individual BCFA. Substrate codes as in Table 1. MixArDebArPG indicate mixed fermentation 1:1:1 (each 0.33% w/v) of arabinan, debranched arabinan, and polygalacturonic acid. **Fig. S4**. Hierarchical clustering between of the different tested substrates as a function SCFA production after 24 h of in vitro simulated colon fermentation with freshly weaned piglet colon content as inoculum. See Table 1 for substrate abbreviations and details on degree of esterification and acetylation for the different substrates. All fermentations were carried out in quadruplicate (with exceptions as stated in Fig. 1).

**Additional file 2. Table S1**: Statistically significant pairwise comparisons in figure 2.

**Additional file 3. Table S2**: Statistically significant correlation pairs between bacterial taxaons and SCFA under the cut-off p < 0.05.

## Data Availability

Not applicable.

## Abbreviations

ASV: Ampicon Sequence Variant
CID: Collision Induced Dissociation
DE: Degree of esterification
DM: Degree of methylation
FOS: Fructooligosaccharide
GC-MS: Gas-Chromatography-Mass Spectrometry
GIT: Gastrointestinal tract
GM: Gut microbiome
GOS: Galactooligosaccharide
HG: Homogalacturonan
HMP: High methylated citrus pectin
HPAEC-PAD: High-Performance Anion-Exchange Chromatography with Pulsed Amperometric Detection
LC-ESI-MS: Liquid Chromatography Electrospray Ionization Mass Spectrometry
LM: Low methoxyl
LMP: Low Methylated citrus Pectin (LMP)
MALDI-TOF: Matrix-Assisted Laser Desorption Ionization Time-Of-Flight
OTU: Operational Taxonomic Unit
PBS: Peptone Buffered Saline
PERMANOVA: Permutational Analysis Of Variance
PME: Pectin methylesterase
PMO: Porcine milk oligosaccharide
POS: Pectic oligosaccharide
RG-I: Rhamnogalacturonan-I
RG-II: Rhamnogalacturonan-II
SBP: Sugar beet pectin
SCFA: Short chain fatty acid
SIM: Selected Ion Monitoring
UHPLC: Ultra High Performance Liquid Chromatograph
