# Supplementary figures and images for: High throughput in vitro characterization of pectins for pig(let) nutrition
Source: Anim Microbiome. 2021 Oct 9;3:69. doi: 10.1186/s42523-021-00129-w (PMC8501679; doi:10.1186/s42523-021-00129-w)

# Supplementary Figure 1

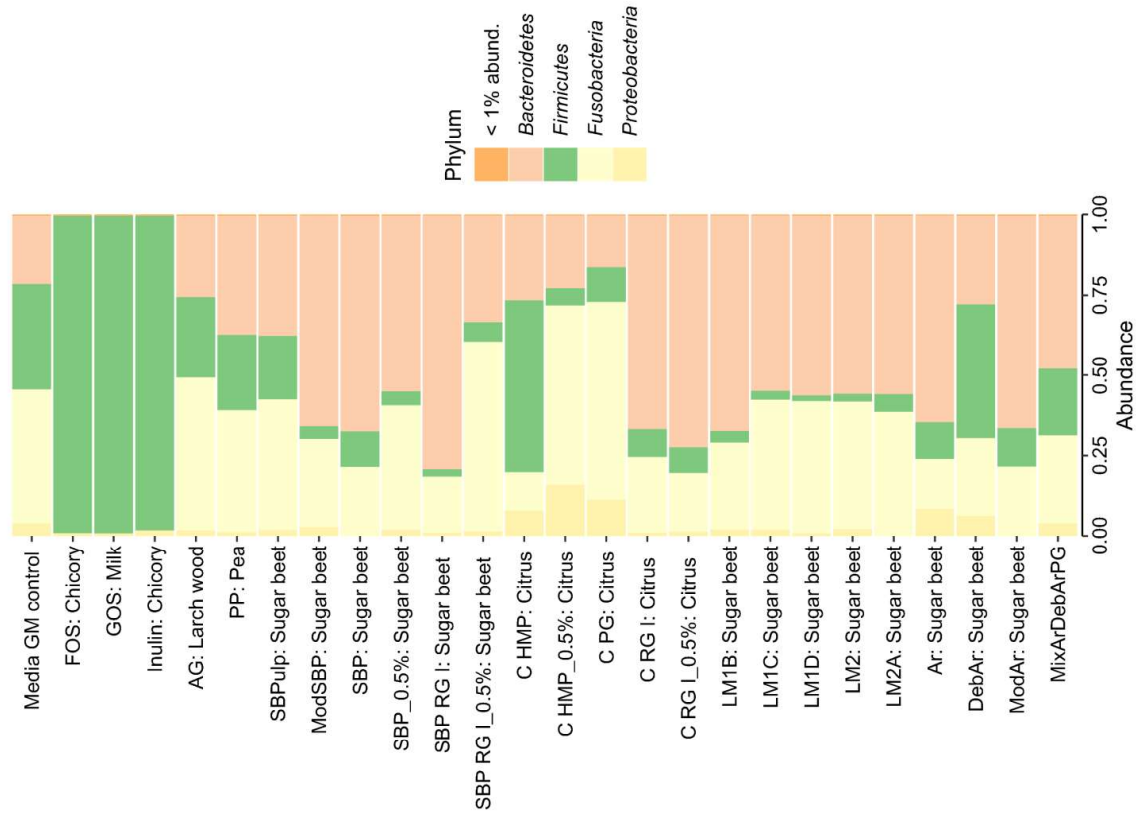





# Supplementary Figure 4

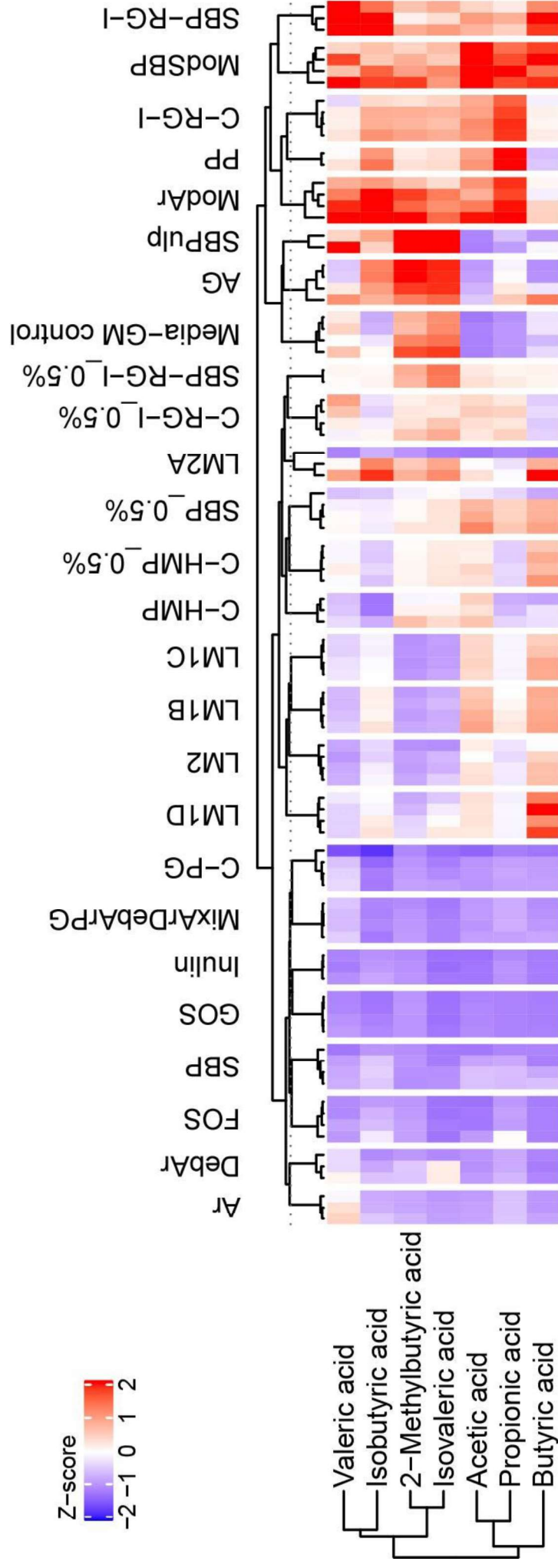

Supplement: Supplementary file 1 — Additional file 1. Fig. S1: Piglet gut microbiota composition (phylum level) as determined by 16S rRNA gene (V3-region) amplicon sequencing after in vitro simulated colon fermentation of different substrates. All fermentations were carried out in quadruplicate (with exceptions as stated in Fig. 1). See Table 1 for substrate abbreviations and details on degree of esterification and acetylation for the different substrates. Fig. S2 Representative microbial taxa of different substrate group relative to media control were plotted in one integrated heatmap (determined by DESeq2 on the summarized lowest classified levels). All fermentations were carried out in quadruplicate (with exceptions as stated in Fig. 1). See Table 1 for substrate abbreviations and details on degree of esterification and acetylation for the different substrates (will be uploaded as high-resolution). Fig. S3. Pairwise comparison of the amount of the branched chain fatty acid (BCFA) produced from different substrates as determined by t test with FDR correction. All fermentations were carried out in quadruplicate (with exceptions as stated in Fig. 1). The symbols *, **, *** represent adjusted P < 0.05, 0.01 and 0.001, respectively. The colour depth of each cell represents the adjusted P value. The values in the square brackets indicate the mean and standard deviation of the individual BCFA. Substrate codes as in Table 1. MixArDebArPG indicate mixed fermentation 1:1:1 (each 0.33% w/v) of arabinan, debranched arabinan, and polygalacturonic acid. Fig. S4. Hierarchical clustering between of the different tested substrates as a function SCFA production after 24 h of in vitro simulated colon fermentation with freshly weaned piglet colon content as inoculum. See Table 1 for substrate abbreviations and details on degree of esterification and acetylation for the different substrates. All fermentations were carried out in quadruplicate (with exceptions as stated in Fig. 1). [file 42523_2021_129_MOESM1_ESM.pdf]
